# Wild gut microbiomes reveal individuals, species, and location as drivers of variation in two critically endangered Hawaiian honeycreepers

**DOI:** 10.7717/peerj.12291

**Published:** 2021-10-28

**Authors:** Maria S. Costantini, Matthew C.I. Medeiros, Lisa H. Crampton, Floyd A. Reed

**Affiliations:** 1 School of Life Sciences, University of Hawaiʻi at Mānoa, Honolulu, Hawaiʻi, United States; 2 Pacific Biosciences Research Center, University of Hawaiʻi at Mānoa, Honolulu, Hawaií, United States; 3 Hawaiʻi Division of Forestry and Wildlife, Hanapepe, Hawaiʻi, United States; 4 Pacific Cooperative Studies Unit, University of Hawaiʻi, Honolulu, Hawaiʻi, United States

**Keywords:** Avian microbiome, Conservation, Microbial ecology, Molecular ecology, Island biology, Tropical birds

## Abstract

**Background:**

The gut microbiome of animals is an important component that has strong influence on the health, fitness, and behavior of its host. Most research in the microbiome field has focused on human populations and commercially important species. However, researchers are now considering the link between endangered species conservation and the microbiome. In Hawaiʻi, several threats (*e.g*., avian malaria and habitat loss) have caused widespread population declines of Hawaiian honeycreepers (subfamily: Carduelinae). These threats can have a significant effect on the avian gut microbiome and may even lead to disruption of microbial function. However, the gut microbiome of honeycreeper in the wild has yet to be explored.

**Methods:**

We collected 13 and 42 fecal samples, respectively, from two critically endangered honeycreeper species, the ʻakikiki (*Oreomystis bairdi*) and the ʻakekeʻe (*Loxops caeruleirostris*). The 16S rRNA gene was sequenced and processed though a MOTHUR-based bioinformatics pipeline. Bacterial ASVs were identified using the DADA2 program and bacterial community analyses, including alpha and beta diversity measures, were conducted using R packages *Phyloseq* and *vegan*.

**Results:**

A total of 8,958 bacterial ASVs were identified from the fecal samples. Intraspecific differences in the gut microbiome among individual birds explained most of the variation present in the dataset, however differences between species did exist. Both species had distinct microbiomes with minimal overlap in beta diversity. ‘Akikiki had a more diverse microbiome compared to ‘akekeʻe. Additionally, small but stastically significant differences in beta diversity also exist between sampling location and sexes in ʻakikiki.

**Conclusion:**

ʻAkikiki and ʻakekeʻe are currently the focus of captive breeding efforts and plans to translocate the two species to other islands are underway. This baseline knowledge will help inform management decisions for these honeycreeper species in their native habitats, on other islands, and in captivity.

## Introduction

Host-associated microbiota—bacteria, fungi, archaea, protists, and viruses—are often critical to the healthy physiological functioning of their host (Kropáčková et al., 2017; Nieves-Ramírez et al., 2018). Additionally, different host organs (*e.g*., gut, skin, vagina) will foster unique microbial communities that possess specific functions. The vertebrate gut microbiome is known to provide a suite of functional benefits for the host, including augmentation of the immune defense against pathogens, digestion and nutrient acquisition, and processing of dietary toxins (reviewed in McFall-Ngai et al., 2013). Despite the known importance of the microbiome throughout the animal world, there is a significant host taxon bias in gut microbiome studies.

Most microbiome research thus far has been on mammals, primarily in humans (Grond et al., 2018). By comparison, the avian microbiome is poorly described and there has only recently been a push to understand the mechanisms that drive microbiome assembly in wild birds (Colston & Jackson, 2016; Hird, 2017; Bodawatta et al., 2021). The microbiomes of avian species are expected to inherently differ from mammalian species because of the route of initial colonization. Mammals are born through their mother’s vaginal canal, which is rich in microbial species (Dominguez-Bello et al., 2010). While there is some evidence that birds may receive maternal transfer of bacteria *in ovo* (Trevelline et al., 2018), it is believed that most of the initial acquisition of microbes happens *via* the environment (*i.e*., the nest and parental crop during feedings; Grond et al., 2017; Chen et al., 2020). Thus, external factors likely a have greater impact on the avian microbiome than the mammalian microbiome.

Hawaiian honeycreepers (*Passeriformes: Fringillidae: Carduelinae*) are a fascinating avian system for ecological- and evolutionary-based questions because of the substantial degree of phenotypic variation due to adaptive radiation within the lineage (Lovette, Bermingham & Ricklefs, 2002). However, despite widespread interest in the group, no research has focused on the gut microbiomes of Hawaiian honeycreepers or any endemic Hawaiian bird species, including two critically endangered honeycreeper species that are endemic to the island of Kauaʻi. The ʻakikiki (*Oreomystis bairdi*) is estimated to have 468 individuals (95% CI [231–916]) and the ʻakekeʻe (*Loxops caeruleirostris*) is estimated to have 945 individuals (95% CI [460–1,547]) remaining in the wild (Paxton et al., 2016). The two species are nearing extinction primarily due to avian malaria (*Plasmodium relictum*) carried by the mosquito vector *Culex quinquefasciatus* (Fortini et al., 2015). Both ʻakikiki and ʻakekeʻe require immediate and drastic conservation actions. However, minimal research has focused on either species and, to some extent, even baseline ecological and life history knowledge is lacking. A survey of the gut microbiome and how it is influenced by internal and external factors in the wild can greatly enhance our knowledge and conservation of these species, while informing our general understanding of the formation of avian microbiomes.

Diet strongly affects microbiome composition across taxa (Pascoe et al., 2017; Youngblut et al., 2019; Teyssier et al., 2020) ʻAkikiki and ʻakekeʻe are both insectivorous species with almost entirely overlapping ranges (Behnke, Pejchar & Crampton, 2016). Nonetheless, the two species forage in different canopy levels and use different foraging techniques (Foster, Scott & Sykes, 2000; Lepson & Pratt, 1997), which may indicate niche partitioning. ʻAkekeʻe are believed to be specialists that forage primarily on one tree species, the ʻōhiʻa lehua (*Metrosideros polymorpha*), by using their unique crossed bill to pry open leaf buds and galls in the terminal branches of the canopy (Lepson & Pratt, 1997). ʻAkikiki glean arthropods off branches in the understory of ʻōhiʻa lehua, as well as several other native tree species (Foster, Scott & Sykes, 2000; VanderWerf & Roberts, 2008). These two different foraging strategies likely result in minimally overlapping diets and, thus, potentially minimally overlapping gut microbial communities. A molecular diet analysis of ʻakikiki and ʻakekeʻe supports the anecdotal evidence that ʻakekeʻe are foraging specialists as their diet is less diverse than that of ʻakikiki (M.S. Costantini, 2020, unpublished data). It is then plausible that ʻakikiki have higher bacterial diversity in their gut microbiomes due to their more generalized diet.

In addition to diet, several other factors both intrinsic (*e.g*., age, breeding condition, sex, evolutionary history) and extrinsic (*e.g*., nesting environment, local prey availability, behavioral interactions) influence microbiome composition (Spor, Koren & Ley, 2011; Grond et al., 2018). In the context of conservation, many of the threats that are causing the decline of honeycreeper populations (*e.g*., habitat degradation, altering prey availability, the introduction of novel species) and the responding management actions (*e.g*., captive breeding) can also affect the host-associated microbiome by altering the available microbial species pool in the environment (Carthey et al., 2020). As a greater diversity of conservation activities (*e.g*., translocation) for both species is considered given their ever-increasing likelihood of extinction, it is imperative that a baseline understanding of their gut microbiomes in the wild is understood. Our goal was to characterize the bacterial community of these two species, specifically by sequencing the 16S rRNA gene. More specifically we aimed to describe the natural patterns in the gut microbiome of ʻakikiki and ʻakekeʻe as they relate to species identity, temporal and spatial sampling patterns, and intraspecific variation of the hosts. Thus, we compared alpha and beta diversity metrics within and between host species at two different locations (one on the periphery of the receding Kauaʻi forest bird range and one in its core).

## Materials and Methods

### Sample collection

**Figure 1:**
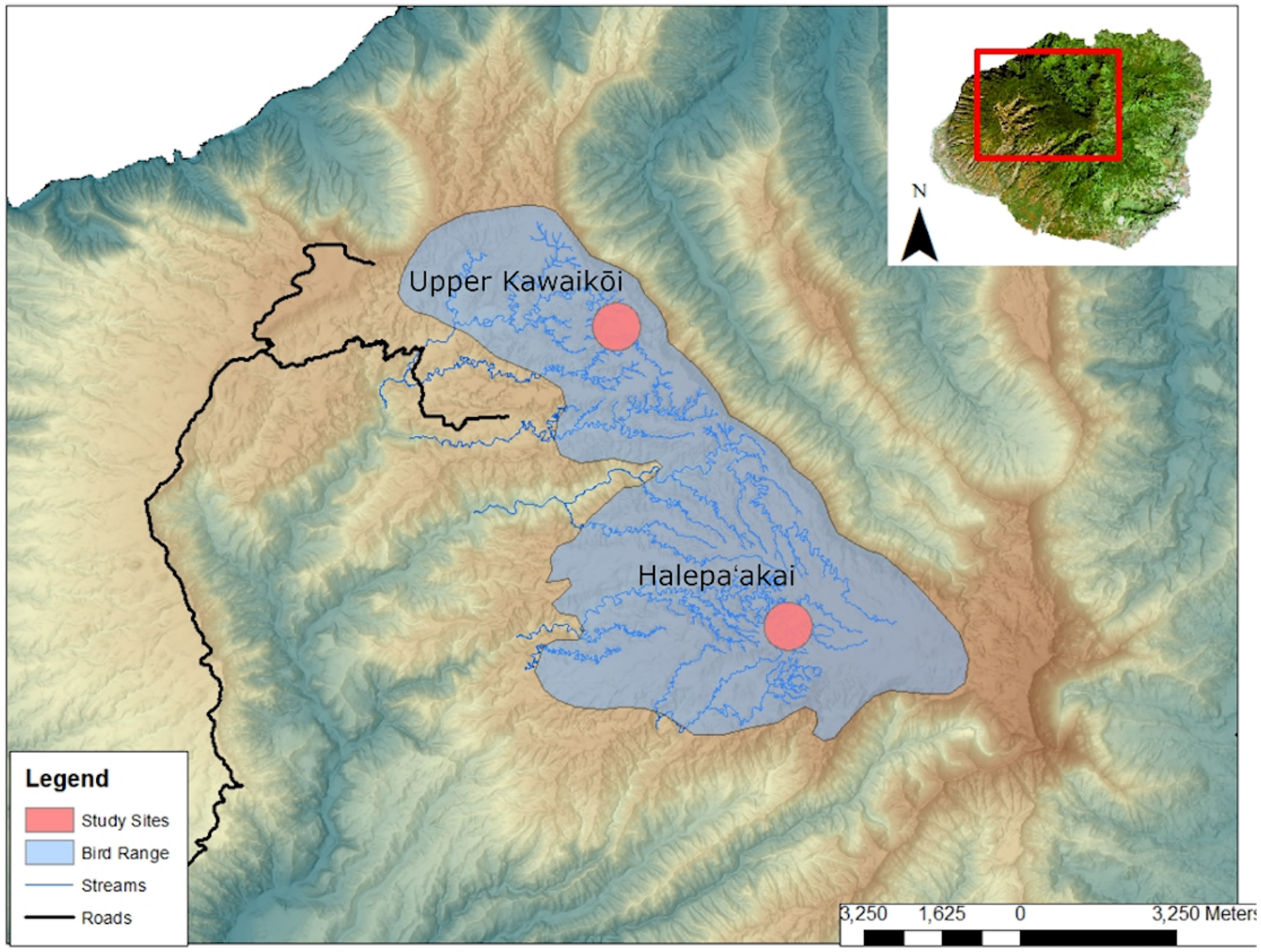
Sampling locations within the remaining endangered forest bird range on the island of Kauaʻi.

We collected a total of 13 fecal samples from ʻakekeʻe (*N* = 13) and 42 fecal samples from ʻakikiki (*N* = 34) between 2016 and 2018 (Table S1). Samples were collected from across the Alakaʻi Plateau on the island of Kauaʻi, Hawaiʻi, representing the remaining range of both species in the wild (Fig. 1). The main field site of the study, Halepaʻakai, is located on the eastern side of the plateau and contains the highest occupancy of both species (Behnke, Pejchar & Crampton, 2016). They are also observed with less frequency, at the Upper Kawaikōi field site. Our study sites on the plateau range from 1,400 m elevation in the east at Halepaʻakai to about 1,200 m in the west at Upper Kawaikōi.

We used fecal samples as a proxy for the gut as they represent the most accurate view of the colon microbiome, short of sacrificing individuals and harvesting the gastrointestinal tract (Videvall et al., 2018). Samples were collected year-round; however, due to the increase in bird activity during the breeding season, most samples were taken between January and June (under IACUC #08-585-7). Upon capture, we placed birds in sterile cloth bags for up to 30 min. Each bag was only used once for a single bird and then retired until it could be washed with bleach and hot water. Fecal samples were directly collected from bags into 1.5 mL Eppendorf tubes containing 100% ethanol and later stored at −20 °C for long-term preservation. We placed unique aluminum USGS leg bands on each individual and recorded age, sex, and morphometric measurements to track body condition.

### DNA extraction and sequencing

We used a Qiagen DNeasy Powersoil Kit to extract DNA from fecal samples by following the manufacturer’s instructions with modification for arthropod diets (Qiagen, Germantown, MD, USA). To remove ethanol from samples before extraction, we conducted two washes of the fecal pellet with RNA/DNA free molecular grade water as described in Grond et al. (2014). Extracted DNA was quantified and quality-controlled using the Invitrogen Qubit 4 Fluorometer (ThermoFisher Scientific, Waltham, MA, USA). Sequencing for bacterial taxa was conducted on the V4 region of the bacterial 16S rRNA gene using forward primer 515F (5′-GTGYCAGCMGCCGCGGTAA-3′) and reverse primer 806R (5′-GGACTACNVGGGTWTCTAAT-3′). The PCR cycling conditions adhered to the Earth Microbiome Project protocol and were as follows: an initial denaturing step at 94 °C for 3 min; followed by 35 cycles of 94 °C for 45 s, 50 °C for 60 s, and 72 °C for 90 s, and then a final extension period at 72 °C for 10 min (Gilbert, Jansson & Knight, 2014). 16S amplicons (PCR products) were purified using Mag-Bind TotalPure NGS beads (Omega Bio-Tek, Norcross, GA, USA) and index tagged to identify the originating bird sample and location and sequenced using the Illumina MiSeq platform with the v3 (2 × 300 cycles) reagent kit. All library preparation and sequencing were conducted at the Advanced Studies in Genomics, Proteomics, and Bioinformatics (ASGPB) facility at the University of Hawaiʻi at Mānoa (Honolulu, HI, USA).

### Data processing and statistical analyses

The University of Hawaiʻi at Mānoa’s C-MAIKI (Center for Microbiome Analysis through Island Knowledge and Investigation) pipeline (Arisdakessian, Cleveland & Belcaid, 2020) for amplicon-based microbiome analysis was used to process samples from raw reads to amplicon sequence variants (ASVs; Callahan, McMurdie & Holmes, 2017). The C-MAIKI pipeline quality-filtered and denoised sequences in the program DADA2, then aligned, filtered, and annotated sequences in the program MOTHUR using the Silva database (Schloss et al., 2009; Callahan et al., 2016; https://www.c-maiki.org/). Potential chimeras were removed with VSEARCH (Rognes et al., 2016) through MOTHUR. Sequences matched to chloroplasts, archaea, and mitochondria were removed from the dataset.

Statistical analyses processing and data visualization was performed in R using the phyloseq, ggplot2, and vegan packages (R Core Team, 2016; Oksanen et al., 2007; McMurdie & Holmes, 2013). The alpha diversity of each gut microbiome was determined by calculating Shannon diversity indices (Table S1) and tested for differences between species, sampling location, sex, and age class using generalized linear models (GLM) with sequencing depth included as a covariate. Significance was tested using the likelihood-ratio chisquare test (‘car’: Anova; Fox & Weisberg, 2019). Differences in gut microbiome beta diversity between certain groups (*i.e*., species, sampling location, sampling season, sampling year, age class, and sex) were visualized using non-metric multidimensional scaling (NMDS) plots with the Bray–Curtis dissimilarity index. To account for differences in sampling depth, we randomly down-sampled (rarefied) to the same read count per sample (16,068 reads per sample). Significance was then tested by calculating non-parametric, permutational multivariate analyses of variance (PERMANOVA; ‘vegan’: *adonis*) with 10,000 permutations.

## Results

Illumina 16S rRNA sequencing yielded 2,667,386 quality-filtered reads (range: 16,068–88,894 sequences per sample; Table S2). We identified a total of 8,958 ASVs after preprocessing the data to remove biologically irrelevant and extremely rare taxa. Rarefaction curves for each individual based on ASV richness indicated that our sequencing depth was sufficient for capturing alpha diversity (Fig. S1). We removed sample “369” from analysis, as it did not appear to amplify.

### Interspecific differences in gut microbiota

**Figure 2:**
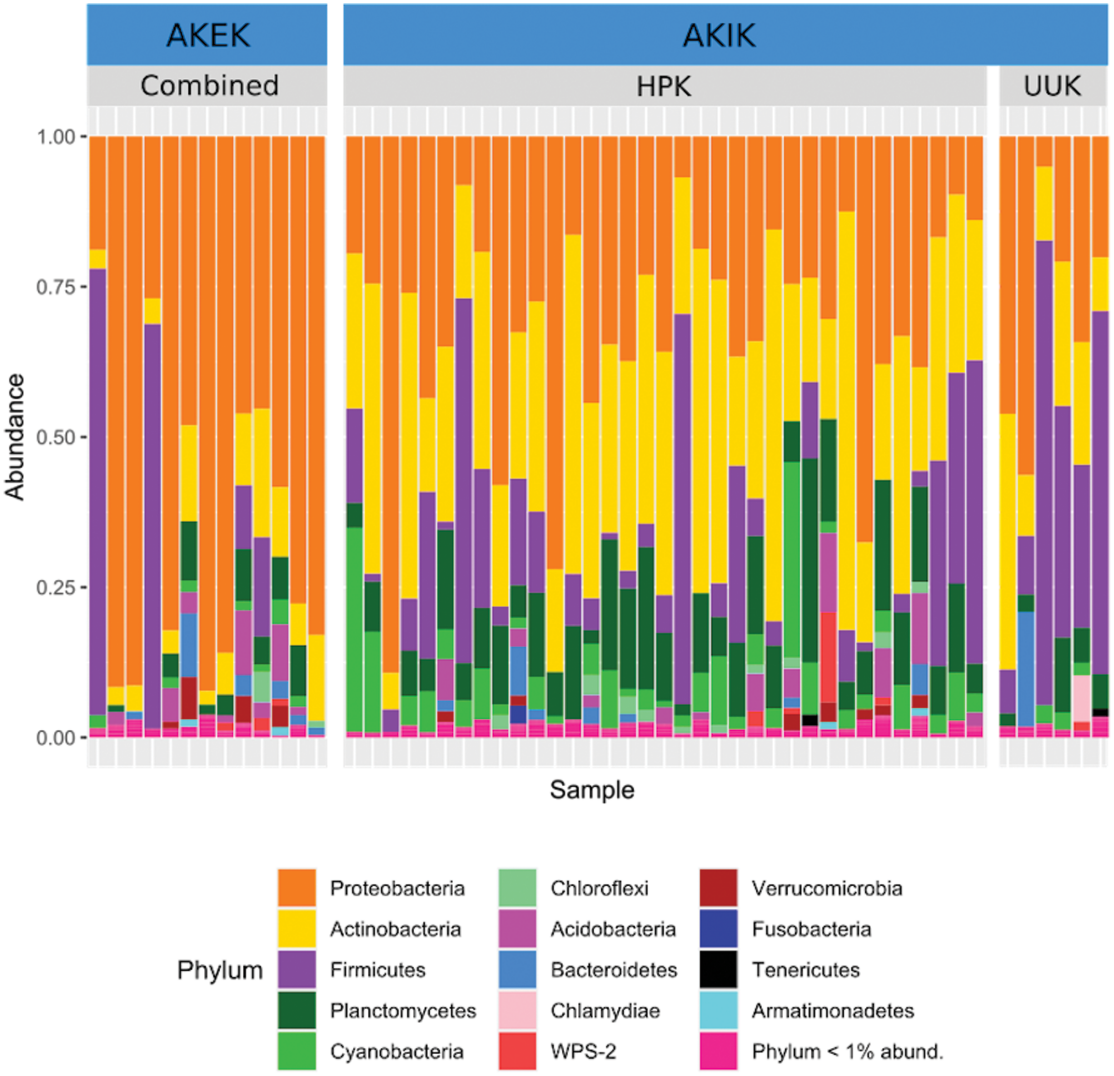
Comparing the relative abundance of bacterial phyla in ʻakikiki and ʻakekeʻe. Phyla that make up less than 1% of the read counts of a sample are grouped together in “Phylum <1% abund.”. Also shown is the comparison of bacterial phyla between the two sampling sites for ʻakikiki. Halepa’akai (HPK) field site represents the core range for the species where occupancy rates are highest, and the habitat is considered near pristine native vegetation. Upper Kawaikōi (UUK) is on the fringe of the ʻakikiki’s present range and has a high density of non-native understory vegetation. ʻAkekeʻe samples are combined by site.

**Figure 3:**
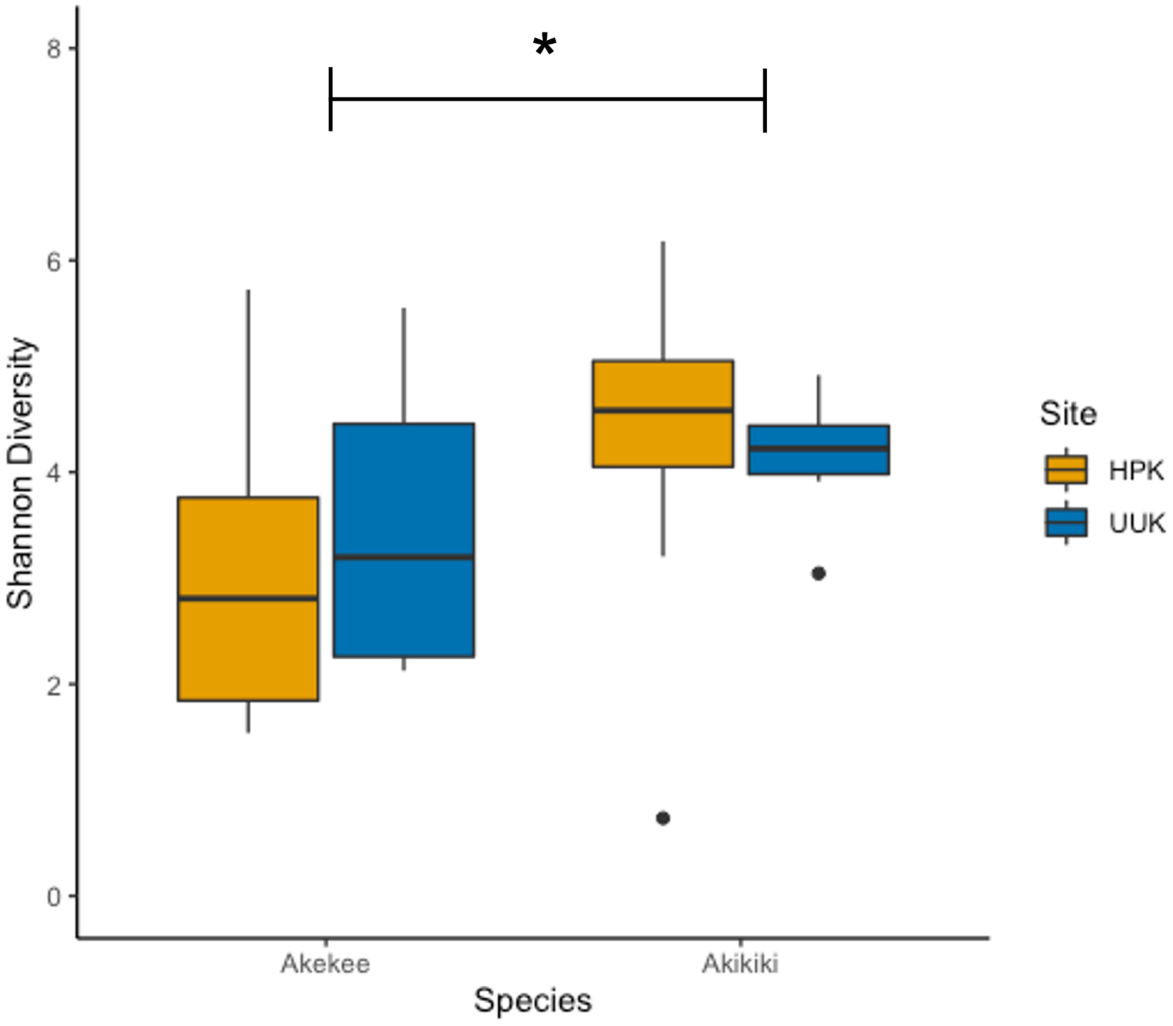
Boxplot of alpha diversity measurements of ʻakikiki and ʻakekeʻe at the two sampling locations. Shannon Index estimates species abundance and evenness of ASVs. There was a significant difference, denoted by an asterick, between species (GLM: *p* = 0.002), but not between sites (only calculated for ʻakikiki due to sample sizes; GLM: *p* = 0.55).

Most variation in the gut microbiome of ʻakikiki and ʻakekeʻe is explained by individual differences. However, the two honeycreeper species have distinct microbial communities from one another, as evidenced by the minimal overlap between species ellipses when plotted with Bray–Curtis distance measurements (Fig. S2; PERMANOVA: df = 1, *R*^2^ = 0.088, *p* = 9.999 × 10^−5^). Despite overall differences in beta diversity, there was still some overlap in taxonomic composition of the two communities. Proteobacteria, Firmicutes, and Actinobacteria generally dominated the gut microbiomes of both ʻakikiki and ʻakekeʻe, but the relative abundances of each phylum differed (Fig. 2). Proteobacteria were more dominant in the ʻakekeʻe microbiome (65.3% average relative abundance) than in that of ʻakikiki (31.1%). In all but two ʻakekeʻe, Proteobacteria made up nearly 50% or greater of all phyla in the gut. In contrast, Proteobacteria was at near-equal levels with Actinobacteria (30.2%) in the ʻakikiki microbiome. Firmicutes were at similar levels in both species and the third most abundant, generally (13% in ʻakekeʻe and 16.7% in ʻakikiki). Another notable difference between the two species was the relatively high occurrence and abundance of Cyanobacteria in ʻakikiki. Cyanobacteria contributed more than 1% of the relative abundance in 87.8% (36/41) of ʻakikiki samples *versus* only 54% (7/13) of ʻakekeʻe samples. Furthermore, in several ʻakikiki individuals, Cyanobacteria comprised a relatively high proportion of the total bacterial abundance (5.4%) compared to in ʻakekeʻe (1.1%). The ʻakikiki gut microbiome had a higher mean alpha diversity than that of ʻakekeʻe (GLM: χ^2^ = 9.46, df = 1, *p* = 0.002; Fig. 3).

### Age differences

To investigate the patterns that drive differences among individuals within a species, we chose to look at only ʻakikiki samples because we had a more robust sample size. Neither alpha diversity (GLM: χ^2^ = 0.07, df = 1, *p* = 0.79) or beta diversity (PERMANOVA: df = 1, *R*^2^ = 0.029, *p* = 0.34) differed between juvenile and adult birds when looking at all pooled samples. We used samples from two ʻakikiki that were sampled as nestlings and resampled as second-year individuals to explore any patterns in the longitudinal acquisition of microbiome members. The bacterial community diversity increased in both birds between nestling and second-year stage (Fig. S3; this was not tested and is only presented anecdotally because of the small sample size, *n* = 2). There was also a notable shift in the composition of the most abundant bacterial phyla. The nestlings’ microbiomes were dominated by Actinobacteria, Proteobacteria, and phyla that comprised less than 5% of the relative abundance of all phyla (Fig. S4). One nestling also possessed a small proportion of the phylum Planctomycetes. Cyanobacteria were present in higher proportions in both second-year individuals, and Firmicutes made up more than 5% of the microbiome in one of the second-year birds.

### Sex differences

**Figure 4:**
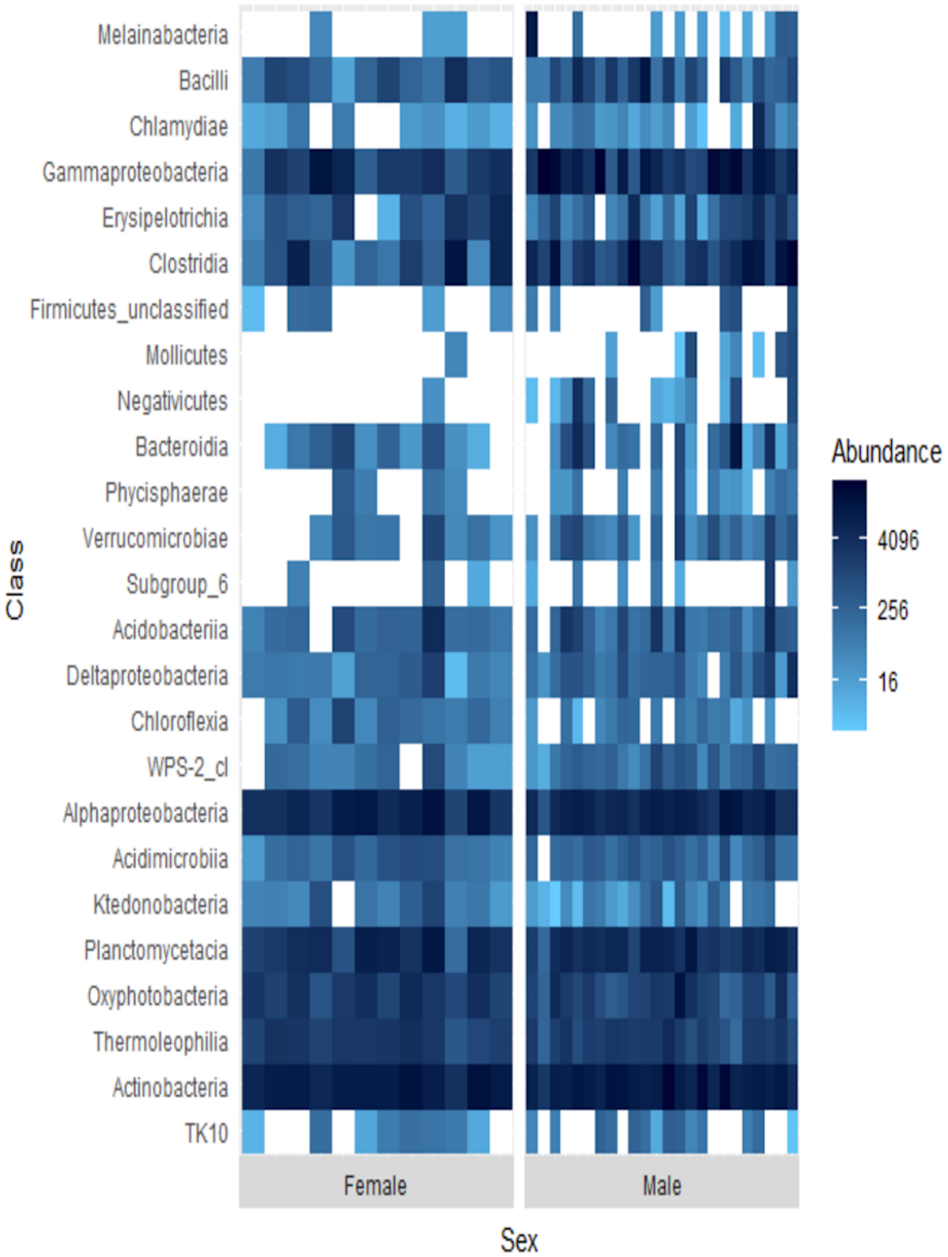
Heatmap showing the relative abundances of the top 25 most abundant bacterial classes in male (*N* = 24) and female (*N* = 12) ʻakikiki. Each box represents an individual sample. Darker blue boxes indicate a greater number of reads of that bacterial class and lighter blue boxes indicate less reads. White boxes indicate that there were no reads of that class in an individual sample.

Alpha diversity levels were similar between female and male ʻakikiki (GLM: χ^2^ = 0.49, df = 1, *p* = 0.48) but differed in beta diversity based on Bray–Curtis dissimilarity (PERMANOVA: df = 1, *R*^2^ = 0.04, *p* = 0.01). One bacterial class, Negativicutes, was distinctly different between the two sexes (Fig. 4). Negativicutes were present in only one female but were found in nine out of the 18 male samples. Other classes, specifically Melainabacteria, Mollicutes, and Phycisphaerae, were uncommon in females but at moderate levels in males.

### Sampling location differences

Temporally, we found no difference in the microbiome structure based on sampling season (classified as “Spring/Summer” or “Winter/Fall”; PERMANOVA: df = 1, *R*^2^ = 0.028, *p* = 0.50) or sampling year (PERMANOVA: df = 1, *R*^2^ = 0.03, *p* = 0.33), but there were spatial differences in the bacterial community structure of ʻakikiki samples between the two sampling sites (PERMANOVA: df = 1, *R*^2^ = 0.05, *p* = 0.0009). Additionally, there was a shift in the relative abundance of key bacterial phyla between the two sites. Actinobacteria comprised fewer of the total phyla in microbiomes of individuals from the Upper Kawaikōi site (Fig. 2). Cyanobacteria and Planctomycetes, two less dominant phyla, were present in higher proportions of ʻakikiki microbiomes from Halepaʻakai. Notably, Cyanobacteria were present in higher proportions within individuals from Halepaʻakai and were present at a greater than 1% abundance in 33 of the 35 (94.3%) ʻakikiki sampled at the location. Only three of the six ʻakikiki sampled at Upper Kawaikōi possessed Cyanobacteria at the same minimal abundance. There were no differences in Shannon diversity values between the Halepaʻakai and Upper Kawaikōi sites (GLM: χ^2^ = 0.35, df = 1, *p* = 0.55; Fig. 3).

## Discussion

The overarching goal of this study was to characterize the wild gut microbiome of two critically endangered Hawaiian honeycreepers and investigate the processes that may influence unique patterns in community composition. Using 16S rRNA amplicon sequencing, we found that the gut microbiomes of ʻakikiki and ʻakekeʻe were distinct from one another, and that ‘akikiki had on average more diverse microbiomes than the ʻakekeʻe. This difference is consistent with the broader foraging and more diverse diet of ʻakikiki relative to ʻakekeʻe, supporting the notion that diet is an essential driver of microbiome community assembly in these species. A broad look at the similarities within ʻakikiki and ʻakekeʻe gut microbiomes reveals that our findings are supported by several wild bird microbiome studies, which generally conclude that Proteobacteria, Firmicutes, and Actinobacteria tend to be the three of the four most dominant phyla in gut microbiomes (Lewis, Moore & Wang, 2017; Kropáčková et al., 2017; Hird et al., 2015; Bodawatta et al., 2018; Grond et al., 2018). ʻAkekeʻe microbiomes are more dominated by Proteobacteria, while Proteobacteria and Actinobacteria, and to a lesser extent, Firmictutes, appear to equally dominate the ʻakikiki gut community. Additionally, both Planctomycetes and Cyanobacteria are more abundant in the ʻakikiki gut microbiome than in that of ʻakekeʻe.

As ʻakikiki are thought to have a more generalized diet, the greater proportional spread of bacterial phyla in ʻakikiki compared to ʻakekeʻe may reflect this dietary difference. Cyanobacteria, which are photosynthetic prokaryotes, are relatively enriched in the ʻakikiki microbiome compared to not only ʻakekeʻe but to other passerine microbiome studies as well (Hird et al., 2015). A potential explanation for their higher proportional abundance may come from ʻakikiki foraging ecology. Cyanobacteria are often the phototrophic partners that live in symbiosis with fungi to make up the complex organisms known as lichen (Nübel, Garcia-Pichel & Muyzer, 1997). ʻAkikiki forage by picking through pieces of moss and lichen to find arthropod prey. Thus, the Cyanobacteria in the ʻakikiki microbiome could either be obtained directly by inadvertent consumption of lichen or indirectly through arthropod prey that had consumed lichen. Another explanation for the abundance of Cyanobacteria in ʻakikiki may be through colonization from water droplets in the moss in which they forage, as many Cyanobacteria live in association with mosses (Rousk, Jones & DeLuca, 2013). Either way, the abundance of Cyanobacteria in the gut likely represents the transient bacterial community and thus, represents more of a reflection of the importance of dietary differences than any functional relevance to the host microbiome.

While differences between species exist in our study, most variation within microbiomes was explained by individual differences. To further investigate potential drivers of individual variation, we looked at environmental and life history characteristics in ʻakikiki only. We found no difference in alpha or beta diversity in the gut microbiome based on sampling season or age class. However, there is suggestive evidence that nestlings and adults harbor distinct microbial communities, based on the repeated measures of two individuals (Figs. S3 and S4). Further investigation into nestling microbiomes will be crucial for these species moving forward, as early disruptions to the microbiome can lead to lifelong issues, such as a reduced immune response to infection (Knutie et al., 2017).

We found weak support for a difference between males and females that may be driven by a few key taxa (Fig. 4). In the current study, we found a higher presence of the bacterial classes Melainabacteria, Mollicutes, Phycisphaerae, and especially, Negativicutes in males than females. Another wild bird study found an association between Negativicutes and male birds (Liu et al., 2020). It is not yet known if this association between the Negativicutes and male birds has any biological relevance. Differences in the gut microbiomes between sexes within bird species can be driven by behavior (*e.g*., differences in foraging ecology) or physiological differences (*e.g*., impacts of sex hormones; Grond et al., 2017).

Lastly, we explored the possibility that sampling habitat affected the gut microbial community of ʻakikiki. Bacterial communities differed significantly in beta diversity between Halepaʻakai and Upper Kawaikōi within the species. Cyanobacteria were present at a level of greater than 1% in the gut microbiome of 94.3% of ʻakikiki samples at Halepaʻakai, but only in 50% of samples from Upper Kawaikōi. Halepaʻakai is considered the last stronghold for the species, where most of the population exists. Furthermore, it is a relatively pristine, undisturbed forest with minor inundation by non-native vegetation (Behnke, Pejchar & Crampton, 2016). In contrast, the sub-population at Upper Kawaikōi was recently discovered in 2018 and represented a small fraction of the total population. The habitat is still dominated by ʻōhiʻa lehua, but the understory has a greater occurrence of non-native and invasive vegetation, including Kahili ginger (*Hedychium gardnerianum*) and strawberry guava (*Psidium cattleianum;* M.S. Costantini, 2018, personal observation). Additionally, for an undetermined reason, many of the trunks of ʻōhiʻa trees in the Upper Kawaikōi site were stripped of moss and lichen (M.S. Costantini, 2018, personal observation).

The difference in relative abundances of Cyanobacteria in ʻakikiki may, again, be explained by diet or foraging differences between birds at the two sites. A less pristine habitat can result in diminished quality and quantity of prey or differences in the environmental microbial community (Trevelline et al., 2019). Interestingly, Cyanobacteria was enriched in the gut microbiome of birds from the more intact site. A recent microbiome study on the American white ibis (*Eudocimus albus*) found that Cyanobacteria significantly decreased in relative abundance with increased urban land cover (Murray et al., 2020). Therefore, the presence and abundance of Cyanobacteria in the avian gut microbiome may indicate the quality of habitat for certain species. Several studies investigating the effect of habitat degradation or land-use change in wild animals have found that animals inhabiting degraded or altered habitats have distinctly different microbiomes, often because of shifting food availability (reviewed in Trevelline et al., 2019). In addition to qualitative changes, hosts living in degraded habitats often face a reduction in microbial alpha diversity compared to conspecifics living in undisturbed habitats (Amato et al., 2013; Barelli et al., 2015; Trevelline et al., 2019). Our results do not show this for ʻakikiki, but follow-up work is necessary with a larger sample size (Fig. 3).

This study represents the first examination of Hawaiian honeycreeper gut microbiomes in the wild. Our goal was to characterize the bacterial communities of two critically endangered species to understand the natural patterns associated with bacterial diversity and composition in the wild. ʻAkikiki and ʻakekeʻe will soon face many conservation challenges as 100% of their suitable habitat is predicted to disappear within this century due to the expansion of avian malaria facilitated by climate change (Paxton et al., 2016; Fortini et al., 2015). Currently, “insurance” populations of both species are established in captivity; however, several studies on other captive animals have demonstrated a distinct shift in the microbial communities when in captivity (Trevelline et al., 2019). This disruption can affect the host in numerous ways. For example, an alteration of the host-associated microbiome can interfere with the acquisition of nutrients or lead to a weakened immune response to pathogens. This challenge is particularly concerning for Hawaiian honeycreepers, whose major threat in the wild is avian malaria (Paxton et al., 2018). Resilience to pathogens conferred by a more robust microbiome may help some individuals combat avian malaria and other diseases.

While the results from this analysis only provide an exploratory survey of the bacterial members of the gut microbiome of ʻakikiki and ʻakekeʻe, it is a necessary first step that must be taken before effective management of the microbiome is possible. Future work will need to focus on determining which microbes are considered symbiotic with the host rather than transient species and how the communities are functionally important. Given the critical connection between the gut microbiome and the proper physiological functioning of the host, it is imperative that the role of the microbiome be considered as conservation management plans move forward with these species.

## Conclusion

We show that the gut microbiomes of ʻakikiki and ʻakekeʻe are distinct from one another and that ʻakikiki, the more generalist forager, had on average more diverse microbiomes than ʻakekeʻe. Both species were dominated by the bacterial phyla Proteobacteria, Firmicutes, and Actinobacteria. For ʻakikiki, sampling site, and to a lesser extent sex, explained a significant degree of variation in the microbial communities between individuals. Age, sampling season, and sampling year did not significantly contribute to microbiome variation.

## Supplemental Information

10.7717/peerj.12291/supp-1 Supplemental Information 1 Rarefaction curves for each individual in the study comparing the bacterial ASV richness to sequencing depth.

10.7717/peerj.12291/supp-2 Supplemental Information 2 Non-metric multidimensional scaling (NMDS) ordination of Bray-Curtis dissimilarity distances for ʻakikiki and ʻakekeʻe. Ellipses represent 90% confidence intervals following a multivariate t-distribution (PERMANOVA: between species, *p* = 9.999 × 10^−5^; between sites for ʻakikiki, *p* = 0.0009).

10.7717/peerj.12291/supp-3 Supplemental Information 3 Alpha diversity measurements for two ʻakikiki individuals as nestlings (N) and second-year (SY) birds. Chao1 values represent nonparametric species richness of ASVs, and the Shannon index estimates species abundance and evenness of AS*Vs*. Each dot is one sample.

10.7717/peerj.12291/supp-4 Supplemental Information 4 Comparing the relative abundance of bacterial phyla of two ʻakikiki individuals sampled at different life stages. Phyla that make up less than 5% of the read counts of a sample are grouped together in “Phylum <5% abund.”. The left panel labeled “N’ represents the birds as nestlings and the right panel labeled “SY” represents the birds as second-year individuals.

10.7717/peerj.12291/supp-5 Supplemental Information 5 Metadata associated with sampled ʻakikiki and ʻakekeʻe. Shannon diversity is shown only once per individual bird.

10.7717/peerj.12291/supp-6 Supplemental Information 6 Accession numbers of 16S amplicon data for ʻakikiki and ʻakekeʻe.

## Additional Information and Declarations

### Data Availability

The following information was supplied regarding data availability: The amplicon sequences are available at NCBI SRA: PRJNA742066; SRR14978318 to SRR14978372.
