# Supplementary figures and images for: Wild gut microbiomes reveal individuals, species, and location as drivers of variation in two critically endangered Hawaiian honeycreepers
Source: PeerJ. 2021 Oct 28;9:e12291. doi: 10.7717/peerj.12291 (PMC8557688; doi:10.7717/peerj.12291)

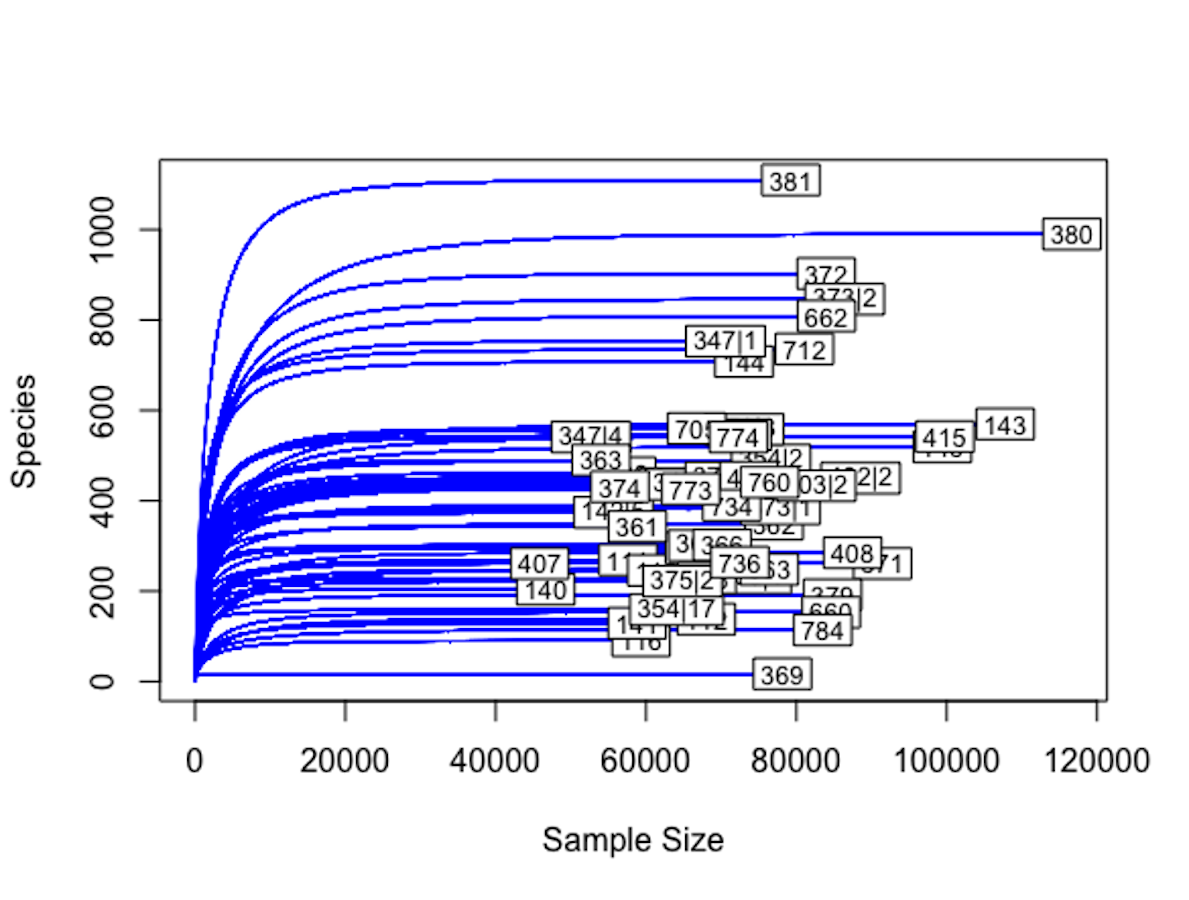

Supplement: Supplemental Information 1 [file peerj-09-12291-s001.png]

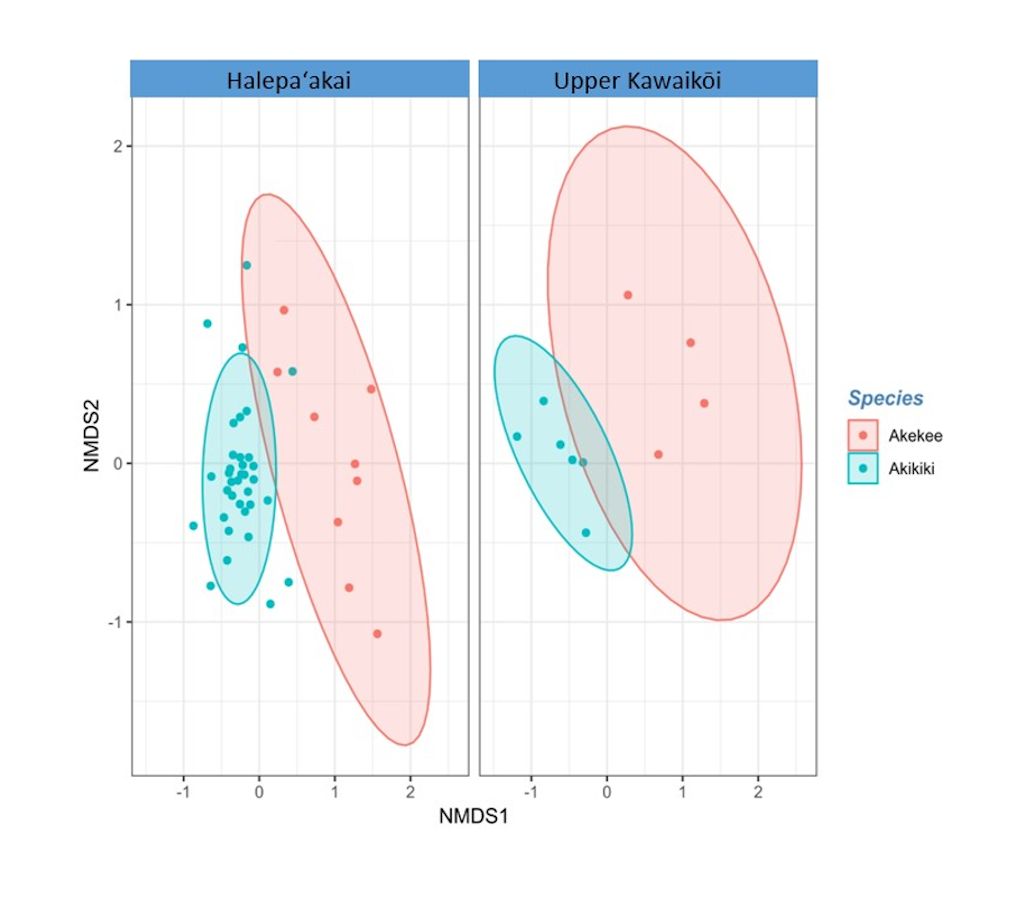

Supplement: Supplemental Information 2 — Ellipses represent 90% confidence intervals following a multivariate t-distribution (PERMANOVA: between species, p = 9.999 × 10^−5^; between sites for ʻakikiki, p = 0.0009). [file peerj-09-12291-s002.png]

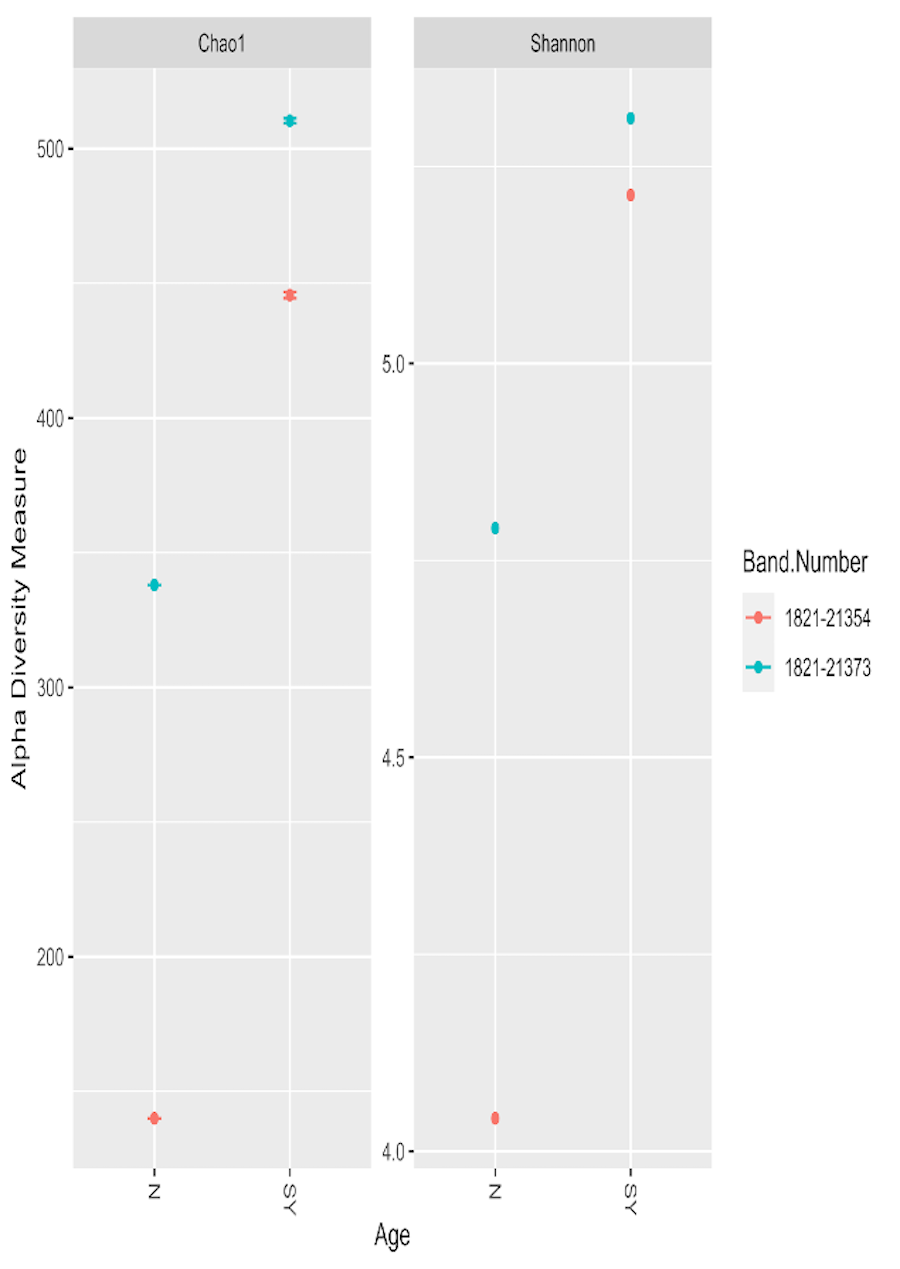

Supplement: Supplemental Information 3 — Chao1 values represent nonparametric species richness of ASVs, and the Shannon index estimates species abundance and evenness of ASVs. Each dot is one sample. [file peerj-09-12291-s003.png]

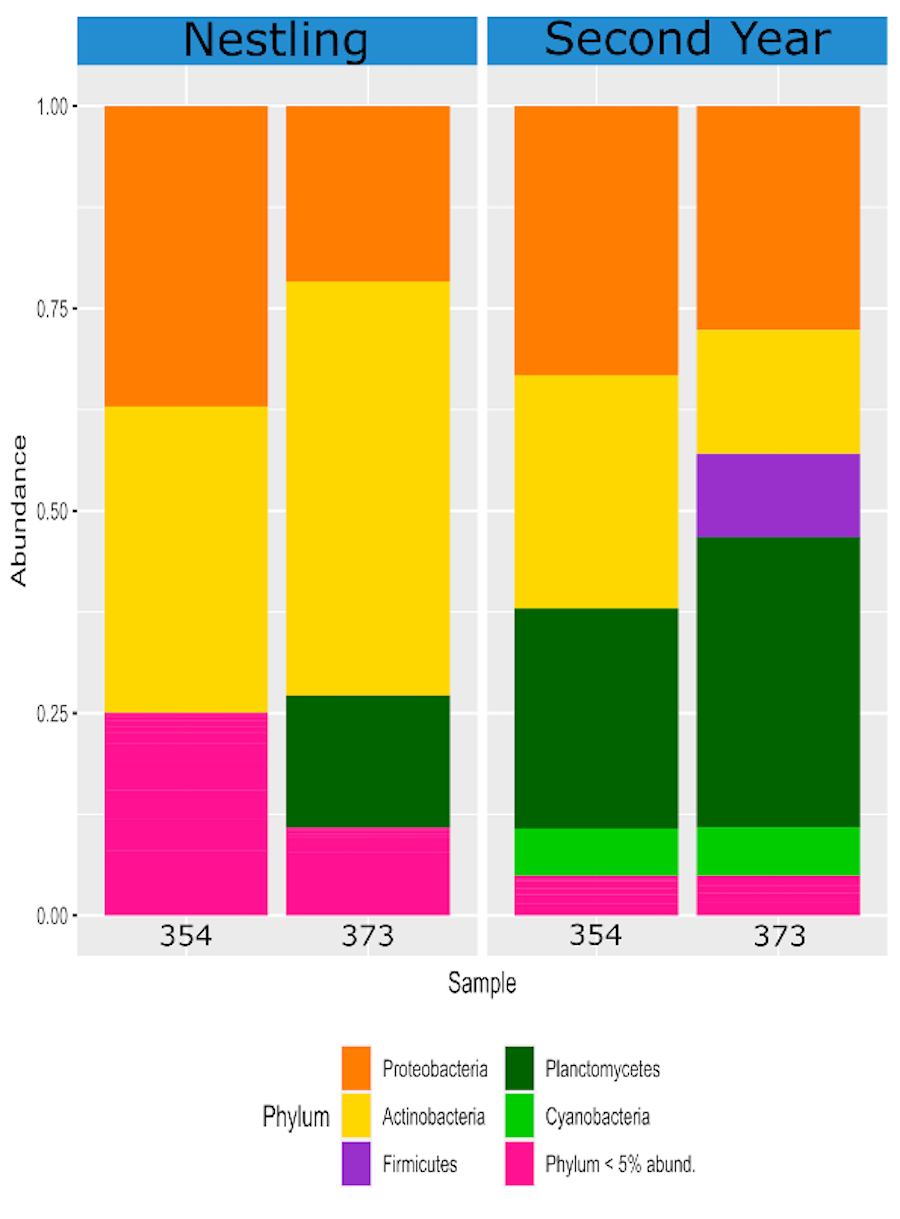

Supplement: Supplemental Information 4 — Phyla that make up less than 5% of the read counts of a sample are grouped together in “Phylum <5% abund.”. The left panel labeled “N’ represents the birds as nestlings and the right panel labeled “SY” represents the birds as second-year individuals. [file peerj-09-12291-s004.png]
