# Supplementary material for: Wild gut microbiomes reveal individuals, species, and location as drivers of variation in two critically endangered Hawaiian honeycreepers
Source: PeerJ. 2021 Oct 28;9:e12291. doi: 10.7717/peerj.12291 (PMC8557688; doi:10.7717/peerj.12291)
Supplement: Supplemental Information 5 — Shannon diversity is shown only once per individual bird. [file peerj-09-12291-s005.docx]

| ID | Band Number | Species | Site | Date Collected | Sex | Age | In Breeding Condition? | Shannon Diversity Index |
| --- | --- | --- | --- | --- | --- | --- | --- | --- |
| 712 | 1821-20712 | ʻakikiki | HPK | 12/13/2017 | Male | ASY | No | 5.85 |
| 774 | 1821-20774 | ʻakikiki | UUK | 11/5/2016 | Male | AHY | No | 4.91 |
| 784 | 1821-20784 | ʻakekeʻe | HPK | 1/31/2017 | Unknown | AHY | No | 2.20 |
| 347\|1 | 1821-21347 | ʻakikiki | HPK | 5/21/2018 | Male | ASY | Yes | - |
| 347\|4 | 1821-21347 | ʻakikiki | HPK | 5/20/2018 | Male | ASY | Yes | 5.11 |
| 347\|3 | 1821-21347 | ʻakikiki | HPK | 4/18/2018 | Male | ASY | Yes | - |
| 347\|2 | 1821-21347 | ʻakikiki | HPK | 1/27/2017 | Male | ASY | No | - |
| 354\|2 | 1821-21354 | ʻakikiki | HPK | 2/7/2018 | Female | SY | No | - |
| 354\|17 | 1821-21354 | ʻakikiki | HPK | 3/17/2017 | Female | N | No | 4.05 |
| 356 | 1821-21356 | ʻakikiki | HPK | 3/17/2017 | Unknown | N | No | 4.70 |
| 360\|3 | 1821-21360 | ʻakikiki | HPK | 3/25/2017 | Female | ASY | Yes | 5.01 |
| 361 | 1821-21361 | ʻakikiki | HPK | 3/26/2017 | Male | SY | No | 4.34 |
| 362 | 1821-21362 | ʻakikiki | HPK | 3/28/2017 | Unknown | N | No | 3.24 |
| 363 | 1821-21363 | ʻakikiki | HPK | 3/28/2017 | Unknown | N | No | 5.09 |
| 366 | 1821-21366 | ʻakikiki | HPK | 4/15/2017 | Female | ASY | Yes | 4.76 |
| 369 | 1821-21369 | ʻakikiki | HPK | 4/9/2017 | Male | ASY | No | - |
| 371 | 1821-21371 | ʻakikiki | HPK | 4/11/2017 | Male | ASY | Yes | 4.05 |
| 372 | 1821-21372 | ʻakikiki | HPK | 4/12/2017 | Male | ASY | Yes | 5.77 |
| 373\|3 | 1821-21373 | ʻakikiki | HPK | 5/6/2018 | Male | SY | No | - |
| 373\|2 | 1821-21373 | ʻakikiki | HPK | 4/13/2017 | Male | N | No | - |
| 373\|1 | 1821-21373 | ʻakikiki | HPK | 4/13/2017 | Male | N | No | 4.81 |
| 374 | 1821-21374 | ʻakikiki | HPK | 4/13/2017 | Unknown | N | No | 4.99 |
| 375\|2 | 1821-21375 | ʻakikiki | HPK | 2/11/2018 | Male | ASY | No | - |
| 375\|1 | 1821-21375 | ʻakikiki | HPK | 4/14/2017 | Male | ASY | Yes | 3.22 |
| 379 | 1821-21379 | ʻakekeʻe | HPK | 4/27/2017 | Female | AHY | Yes | 2.96 |
| 380 | 1821-21380 | ʻakekeʻe | HPK | 4/27/2017 | Male | ASY | Yes | 5.73 |
| 381 | 1821-21381 | ʻakikiki | HPK | 4/25/2017 | Female | SY | Yes | 6.18 |
| 402\|2 | 1821-21402 | ʻakikiki | UUK | 2/24/2018 | Male | ASY | Yes | 4.26 |
| 403\|2 | 1821-21403 | ʻakikiki | UUK | 2/24/2018 | Male | ASY | No | 3.91 |
| 407 | 1821-21407 | ʻakikiki | UUK | 2/27/2018 | Female | ASY | Yes | 3.05 |
| 408 | 1821-21408 | ʻakekeʻe | UUK | 2/27/2018 | Unknown | AHY | No | 2.13 |
| 411 | 1821-21411 | ʻakikiki | UUK | 3/7/2018 | Male | ASY | Yes | 4.50 |
| 415 | 1821-21415 | ʻakikiki | UUK | 3/26/2018 | Unknown | SY | No | 4.19 |
| 300\|2 | 2280-09300 | ʻakikiki | HPK | 3/29/2018 | Female | ASY | Yes | - |
| 300\|1 | 2280-09300 | ʻakikiki | HPK | 1/30/2017 | Female | ASY | No | 4.52 |
| 644 | 2280-37644 | ʻakikiki | HPK | 4/17/2017 | Female | AHY | Yes | 5.30 |
| 660 | 2280-37660 | ʻakekeʻe | UUK | 2/24/2018 | Unknown | AHY | No | 2.30 |
| 662 | 2280-37662 | ʻakekeʻe | UUK | 2/24/2018 | Unknown | AHY | No | 5.55 |
| 663 | 2280-37663 | ʻakekeʻe | UUK | 2/24/2018 | Unknown | AHY | No | 4.09 |
| 705 | 2280-37705 | ʻakekeʻe | HPK | 3/26/2017 | Male | AHY | No | 5.25 |
| 734 | 2280-37734 | ʻakikiki | HPK | 1/20/2018 | Female | ASY | No | 4.24 |
| 736 | 2280-37736 | ʻakikiki | HPK | 1/21/2018 | Unknown | SY | No | 4.55 |
| 760 | 2280-37760 | ʻakekeʻe | HPK | 4/7/2018 | Female | ASY | Yes | 3.76 |
| 773 | 2280-37773 | ʻakikiki | HPK | 5/12/2018 | Unknown | HY | No | 3.20 |
| 106 | 2631-36106 | ʻakikiki | HPK | 1/20/2018 | Male | ASY | No | 4.14 |
| 111 | 2631-36111 | ʻakikiki | HPK | 1/25/2018 | Unknown | SY | No | 4.58 |
| 112 | 2631-36112 | ʻakekeʻe | HPK | 1/25/2018 | Unknown | AHY | No | 1.54 |
| 116 | 2631-36116 | ʻakikiki | HPK | 2/7/2018 | Male | ASY | No | 0.73 |
| 118 | 2631-36118 | ʻakekeʻe | HPK | 2/7/2018 | Unknown | AHY | No | 1.84 |
| 140 | 2631-36140 | ʻakekeʻe | HPK | 4/20/2018 | Male | AHY | Yes | 2.81 |
| 141 | 2631-36141 | ʻakekeʻe | HPK | 4/20/2018 | Male | AHY | Yes | 1.61 |
| 142\|5 | 2631-36142 | ʻakikiki | HPK | 5/7/2018 | Female | SY | Yes | 4.85 |
| 143 | 2631-36143 | ʻakikiki | HPK | 5/12/2018 | Male | SY | Yes | 3.60 |
| 144 | 2631-36144 | ʻakikiki | HPK | 5/28/2018 | Male | ASY | No | 5.86 |
| 145 | 2631-36145 | ʻakikiki | HPK | 5/28/2018 | Unknown | HY | No | 3.68 |
